# Comb-locked Lamb-dip spectrometer

**DOI:** 10.1038/srep27183

**Published:** 2016-06-06

**Authors:** Davide Gatti, Riccardo Gotti, Alessio Gambetta, Michele Belmonte, Gianluca Galzerano, Paolo Laporta, Marco Marangoni

**Affiliations:** 1 Istituto di Fotonica e Nanotecnologie-Consiglio Nazionale delle Ricerche, P.za L. da Vinci 32, 20133 Milano, Italy; 2 Dipartimento di Fisica - Politecnico di Milano, Via Gaetano Previati 1/C, 23900 Lecco, Italy; 3 Oclaro Inc. - via F. Fellini, 4, 20097 San Donato Milanese, Italy

## Abstract

Overcoming the Doppler broadening limit is a cornerstone of precision spectroscopy. Nevertheless, the achievement of a Doppler-free regime is severely hampered by the need of high field intensities to saturate absorption transitions and of a high signal-to-noise ratio to detect tiny Lamb-dip features. Here we present a novel comb-assisted spectrometer ensuring over a broad range from 1.5 to 1.63 μm intra-cavity field enhancement up to 1.5 kW/cm^2^, which is suitable for saturation of transitions with extremely weak electric dipole moments. Referencing to an optical frequency comb allows the spectrometer to operate with kHz-level frequency accuracy, while an extremely tight locking of the probe laser to the enhancement cavity enables a 10^−11^ cm^−1^ absorption sensitivity to be reached over 200 s in a purely dc direct-detection-mode at the cavity output. The particularly simple and robust detection and operating scheme, together with the wide tunability available, makes the system suitable to explore thousands of lines of several molecules never observed so far in a Doppler-free regime. As a demonstration, Lamb-dip spectroscopy is performed on the P(15) line of the 01120-00000 band of acetylene, featuring a line-strength below 10^−23^ cm/mol and an Einstein coefficient of 5 mHz, among the weakest ever observed.

The saturated absorption regime has been recognized since the 70’s as an extremely powerful approach to push the frequency precision of an optical spectrometer far below the limit set by Doppler broadening^1^. As it is well known, when the frequency of two counter-propagating beams lies at the centre of a given transition, the two beams are allowed to interact with a same class of molecules, namely those sharing zero velocity along the propagation direction: in this condition, if the intensity of one of the beams is high enough to depopulate the ground level of the transition, the second beam experiences a decreased absorption that manifests at the centre of the absorption spectrum as a dip, as commonly called Lamb dip^2^. With a typical width of ∼1 MHz set by pressure and transit-time broadening, i.e. 2–3 orders of magnitude below the Doppler limit, and with a contrast up to 10% of the linear absorption, accessing Lamb dips may improve the precision of line centre frequency determinations by 1–2 orders of magnitude for a given signal-to-noise ratio of the measurement. Narrow dips are also favourable to get rid of spectral baseline distortions caused by etaloning effects, which ultimately impair the fit of absorption spectra acquired over large frequency scans. Moreover, dips are insensitive to the asymmetry of Doppler-broadened absorption profiles due to the speed-dependent impact of intermolecular collisions^3^, thus suppressing a potential source of systematic errors in Doppler-limited measurements.

Notwithstanding undisputable advantages, our knowledge of the energy structure of molecules^4^ mostly reflects spectroscopic data acquired in a linear absorption regime. Before the invention of optical frequency combs^5^, which made spectroscopic determinations absolutely traceable, the additional precision given by saturated spectroscopy was traded off by the absence of tools enabling a comparable level of accuracy. The advent of combs revitalized the Doppler-free regime and prompted the absolute calibration of line centre frequencies of several molecules in the visible^6^,^7^, near-infrared^8^,^9^ and mid-infrared range^10^,^11^,^12^,^13^. However, extended line surveys with retrieval of the relevant band spectroscopic constants have been performed only on quite intense lines, such as for ^13^C_2_H_2_ (10100-00000 band^14^,^15^,^16^), C_2_H_2_ (10100-00000 band^17^,^18^, hot 10110-00010 and 10101-00001 bands^19^), NH_3_ (1010-0000 and 1002-0000 bands^20^) and ^18^H_2_O (101-000 band^21^) in the near-infrared, CO_2_ (laser bands at 9 and 10 μm^22^) and N_2_O (100 ← 000 band^23^) in the mid-infrared. For less intense lines, a linear absorption regime has been preferred^24^,^25^, taking advantage of the extremely linear and repeatable frequency axis of the comb over wide spectral ranges. The major reason for the rather limited deployment of Doppler-free spectroscopy remains the difficulty to achieve at once the intensity level required to saturate the transition and the absorption level needed for the dip, which is a fraction of the absorption, to emerge from the noise. In the near-infrared, where the line-strengths are rather small and typically below 10^−22^ cm/mol, the adoption of a high-finesse cavity may serve both purposes, since the finesse effectively multiplies both intra-cavity power and absorption length.

Among cavity-enhanced techniques, cavity-ring-down-spectroscopy (CRDS)^26^,^27^ has the merit of a substantially simple setup and of a strong immunity against the laser frequency noise. Such noise decreases the cavity throughput but does not necessarily lead to a strong degradation of the signal-to-noise ratio on the exponentially decaying waveform, at least as long as the cavity throughput favourably compares with the detector noise. This was brilliantly demonstrated by Campargue and Kassi^28^, with a noise limit at a 6∙10^−11^ cm^−1^ level on the single ring-down event in spite of 250-Hz cavity resonances and of a 2-MHz laser linewidth. Lamb dips by CRDS have been observed on NO_2_ lines near 0.8 μm^29^, on water lines near 1.4 μm^30^ and on ^17^O^12^C^16^O at natural abundance near 2.3 μm^31^. Recently, by adoption of a particularly evolved CRDS apparatus seeded by a an ultra-stable laser, CO_2_ lines as weak as 5·10^−25^ cm/mol with Einstein coefficients down to 7 mHz were saturated and exploited to revisit the spectroscopic constants of the CO_2_ 30013 ← 00001 band^32^. Interestingly, the noise level of 1.7·10^−11^ cm^−1^ over a 7-minute-long measurement remained nearly 2 orders of magnitude above the limit of detection of the same spectrometer in a linear absorption regime, equal to 5∙10^−13^ cm^−1^ over 1 s^33^. This derives from two main issues of Doppler-free CRDS spectroscopy: i) the shorter ring-down time that occurs at the centre of the absorption line, where the dip takes place, and ii) the failure of the exponential function for the fit of ring-down decays in a saturated regime. This makes the fit more susceptible to fluctuating conditions such as the optical power coupled into the cavity at the beginning of the decay. Introducing a second decay constant in the fit function improves the fit quality and also helps in normalizing the absorption against the empty-cavity losses, as demonstrated by Galli *et al*.^34^, yet it may increase the error bar due to the correlation between the two decay constants.

An alternative and particularly elegant approach to Doppler-free spectroscopy that does not suffer from the above trade-offs is represented by Noise-Immune Cavity-Enhanced Optical-Heterodyne Molecular Spectroscopy (NICE-OHMS). NICE-OHMS is a smart combination of tight laser-to-the-cavity locking, achieved through the Pound-Drever-Hall (PDH) technique^35^, with a frequency-modulation-spectroscopy approach where sidebands are placed coincident with the neighbouring cavity modes. The immunity of NICE-OHMS against laser-cavity frequency noise was demonstrated by Ma *et al*.^36^ almost two decades ago with the saturation of a very weak overtone line of CH_2_D at 1064 nm, with a nearly shot-noise-limited sensitivity of 10^−14^ cm^−1^Hz^−0.5^. A similar value has been recently approached even in a Doppler-broadening regime on the P(11) line of C_2_H_2_^37^, yet recurring, as in the first demonstration, to an ultra-narrow seed laser with very poor frequency tunability to achieve a tight lock between laser and cavity. With more versatile and tunable laser sources such lock and thus the sensitivity limit was found to be degraded by one or even two or three orders of magnitude^38^,^39^, which explains why NICE-OHMS failed to be applied to broad line surveys in a saturated regime.

This paper describes a versatile cavity-enhanced optical spectrometer for Lamb-dip spectroscopy over the range from 1.5 to 1.63 μm, featuring frequency accuracy at the kHz-level and a limit of detection below 2.5∙10^−11^ cm^−1^ over a typical 200-s acquisition time. The accuracy of the spectrometer is based on the referencing to an Er:fiber frequency comb whereas the sensitivity on an extremely tight laser-cavity PDH lock that brings the relative amplitude noise at the cavity output below 10^−3^ in a direct detection mode, without recurring to any wavelength- or frequency-modulation-spectroscopy approach and thus with a very simple scheme. A cavity finesse in excess of 100000 allows Lamb-dip spectroscopy to be performed also on weak transitions, whereas the possibility to vary the intra-cavity field intensity by more than two orders of magnitude up to the kW/cm^2^ level enables saturation also of highly symmetric vibration modes with poor electric dipole moment. This is experimentally demonstrated on the P(15) line of the 01120-00000 band of C_2_H_2_, which features a line-strength below 10^−23^ cm/mol and an Einstein coefficient of only 5 mHz. At the opposite side of the application range, saturation is also demonstrated for the P(30), P(28) and P(26) lines of the well characterized 10100-00000 band of C_2_H_2_, with line strengths of 10^−22^ cm/mol and Einstein coefficients 1000 times higher. Thousands of lines in different bands for several molecules may be found within these wide boundary conditions, which makes the system an unprecedented tool for Doppler-free spectroscopy in a spectral region of extreme spectroscopic interest.

## Experimental Setup

**Figure 1:**
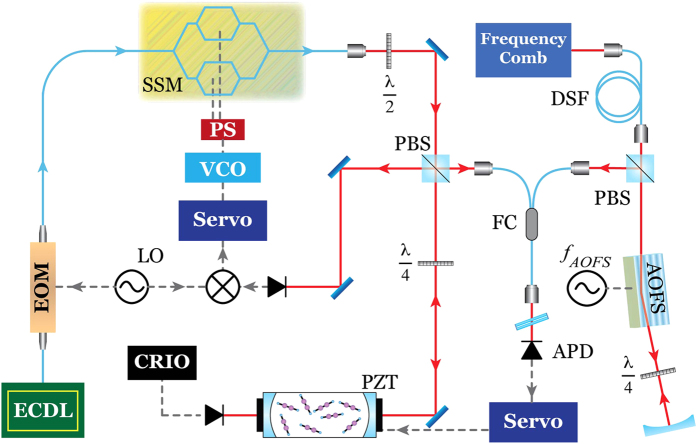
Experimental setup. ECDL: extended-cavity diode laser; EOM: electro-optical modulator; SSM: single-sideband modulator; VCO: voltage-controlled oscillator; PS: hybrid 90° phase-shifter; PBS: polarizing beam splitter; PZT: piezoelectric transducer; FC: fiber coupler; DSF: dispersion-shifted fiber; APD: avalanche photodetector; AOFS: acousto-optical frequency shifter; CRIO (Compact Reconfigurable Input/Output): FPGA-based programmable automation controller. Blue and red lines refer to fiber and free-space paths, respectively, while dashed grey lines to electrical connections.

The layout of the spectrometer is sketched in Fig. 1. An extended-cavity-diode-laser (ECDL) with broad tunability from 1.5 to 1.63 μm (Toptica DL pro) is locked with a Pound-Drever-Hall (PDH) approach to a stainless-steel cavity with a finesse >100000. PDH locking is achieved through a single proportional-integral-derivative (PID) servo that provides external correction to the laser frequency through a voltage-controlled-oscillator (VCO) driven single-sideband modulator (SSM), that acts as an ultrafast frequency actuator, as described in ref. 40. An output voltage dynamics of ±3.5 V for the PID and a frequency-to-voltage conversion of 22 MHz/V for the VCO allow the laser to remain steadily locked upon tuning the cavity mode by up to ±75 MHz, thus far in excess of a typical Lamb-dip spectral width. The control bandwidth, as high as 5 MHz, squeezes the residual laser-cavity linewidth down to 4 mHz, strongly suppressing intensity noise at the cavity output caused by frequency-to-amplitude conversion of cavity and laser frequency noise.

The above conditions make a simple cavity-enhanced direct-detection approach of great attractiveness. The power transmitted by the cavity is measured by a commercial detector with variable gain from 10^3^ to 10^7^ V/W (Thorlabs PDB150C). This ensures an electrical signal of few Volts, well above the quantization noise of the 16-bit FPGA-based board used for data acquisition, whatever is the optical power from below 1 μW to the maximum achieved value of about 100 μW. The latter case, with a cavity length of 50 cm, mirrors with losses of 1.1∙10^−5^ and 1-m radius of curvature, corresponds to an intra-cavity field intensity in excess of 1.5 kW/cm^2^, which is sufficient to provide saturation parameters of few % for transitions with Einstein coefficients down to few mHz.

Absolute calibration of the frequency scans is obtained through the referencing of the cavity-locked probe laser to a frequency comb synthesized by a 100-MHz Er:fibre oscillator (Toptica FFS) with repetition- and carrier-envelope-offset-frequencies stabilized to a primary clock through a GPS-referred Rubidium oscillator. The comb is made to beat on an avalanche photodetector with a fraction of the ECDL and their beat note is stabilized by slow feedback (700 Hz bandwidth) to a piezoelectric transducer acting on the cavity length and thus on the cavity mode that the ECDL is locked to. In this way the cavity mode (which the ECDL is locked to) is forced to lie at a given offset from the nearest comb mode. In order to perform comb-calibrated scans of the laser frequency, the comb undergoes a double pass in an acousto-optic frequency shifter (AOFS) before superimposing the ECDL: the AOFS provides a single-pass diffraction efficiency higher than 75% in the 75–95 MHz RF range, giving rise to an optical tuning range of 40 MHz for the comb and thus for the ECDL. Frequency scans are thus accomplished by means of a RF synthesizer that drives in a controlled way the input signal of the AOFS. In order to maintain a stable frequency offset between comb and ECDL over multiple scans, the beat note signal at the input of the feedback loop must exhibit a good signal-to-noise ratio (SNR): experimentally, by counting the beat note during the scans and looking at its deviations, we found a 30-dB SNR at a resolution bandwidth of 10 kHz to be necessary to push rms deviations below 0.2 kHz. In our case, due to the relatively low repetition frequency and average optical power of the comb, such threshold was not reached over the entire tuning range of the ECDL using the comb supercontinuum output. We then exploited a second 1.55-μm comb output and got it spectrally broadened in a simple dispersion-shifted telecom fiber over a region much narrower than an octave, roughly from 1.4 to 1.7 μm, in order to enhance the average power per comb mode over the rather large tuning range of the ECDL (1.5–1.63 μm).

## Results and Discussion

**Figure 2:**
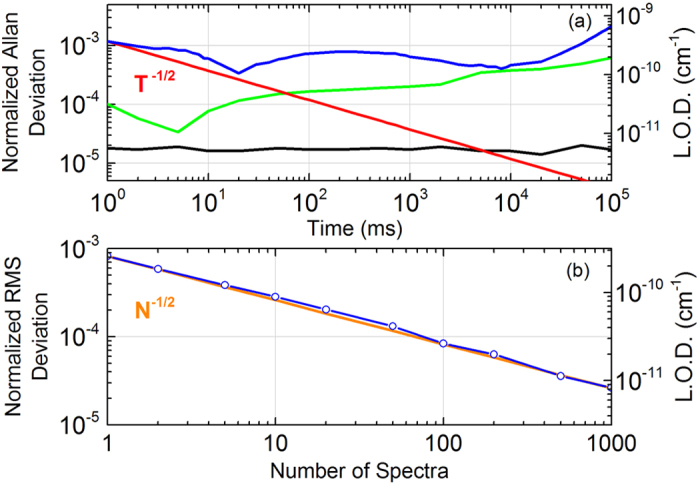
(**a**) Normalized Allan deviation (left) and corresponding limit of detection (right) calculated for the power transmitted from the cavity (blue curve), as compared to that at the entrance of the cavity (green curve) and to the noise background given by the photodetector (black line). (**b**) rms value of the residuals obtained from the fit of N-times averaged blank spectra to a linear function (blue curve), as compared to the inverse square-root law expected for a normal noise distribution (yellow curve).

In a cavity-enhanced direct-detection regime the minimum detectable absorption *α*_*min*_ is related to the laser intensity noise at the cavity output through the simple formula:


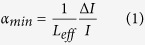


which derives from a first-order approximation of the Lambert-Beer law. In Eq. (1) Δ*I/I* is the root-mean-square value of the relative intensity noise over a given measurement time and *L*_*eff*_ is the effective interaction length. Under weak absorption conditions this is in turn proportional to the geometrical length *L* and to the finesse *F* of the cavity, by the relation *L*_*eff*_ = 2∙*L∙F*/π^41^. Increasing *F* and *L* is thus beneficial for the sensitivity of the spectrometer provided that the intensity noise term is preserved. This is in practice a challenge since the higher is *L*_*eff*_ the steeper is the slope of the frequency-to-amplitude response given by the cavity modes, with the consequence that any residual frequency noise between laser and cavity may severely impact on Δ*I*/*I* and thus on *α*_*min*_. In our case, thanks to a very tight laser-cavity locking, such a detrimental noise conversion is strongly inhibited. This is shown in Fig. 2(a), where an Allan plot (blue curve) is reported for Δ*I*/*I* as a function of the averaging time for times longer than 1 ms. Below 1 ms the data are not relevant since they correspond to measurement bandwidths that can’t be exploited experimentally since they are in excess of the 700 Hz-bandwidth of the cavity-comb feedback loop. Moreover, below 1 ms, it is more difficult to evaluate the impact of residual laser-cavity frequency noise since the cavity acts as a low pass filter (with a pole at half the 3-kHz-wide cavity mode). The Allan deviation amounts to 8∙10^−4^ at the 10-ms averaging time used in the measurements, it decreases till 4·10^−4^ at 20 ms while remaining almost constant up to 10 s. This is the signature of a noise dominated by a Flicker contribution, which is mostly due to residual frequency noise between cavity and laser. This is confirmed by the green trace reported in the figure, which refers to a measurement performed before the cavity where Δ*I*/*I* is mostly affected by laser intensity noise: this remains a factor greater than 4 below the frequency noise contribution over the typical 2-s-long measurement time taken by each frequency scan. On the other hand the detector noise remains more than an order of magnitude below whatever is the gain setting (black line). To improve the limit of detection in a system dominated by Flicker noise it is beneficial to speed up the acquisition of a single spectrum while averaging over many subsequent spectra: in our case, due to the relatively slow cavity-comb feedback loop, optimum conditions were found by stepping the laser frequency at every 20 ms, by letting the cavity-locked laser to stabilize against the comb within the first 10 ms, then acquiring and averaging the cavity output power over the last 10 ms. This implies a 100-spectral-points spectrum to be acquired in 2 s. Figure 2(b) reports the normalized rms deviation of residuals for N-times averaged blank spectra acquired in those conditions and fitted with a linear baseline. At N = 1 the deviation amounts to 8·10^−4^, in perfect agreement with the Allan plot over the corresponding time window; at increasing N, it decreases with the square root of N as expected for a normal noise distribution, attaining a value of 2.5·10^−5^ at N = 1000, i.e. 2000 s. This corresponds to a detection limit of 7.8∙10^−12^ cm^−1^, which is, to the best of our knowledge, the lowest ever demonstrated in a direct-detection cavity-enhanced regime starting from a widely tunable laser with linewidth in excess of 100 kHz. The inverse square-root law shown in Fig. 2(b) was found to hold for frequency scans below 30 MHz that are typical of the Lamb-dip regime thanks to the absence in the setup of parasitic cavities with comparable free-spectral-range.

Precision and accuracy of the spectrometer were tested on three well-characterized^17^,^18^ sub-Doppler lines of the 10100-00000 band of acetylene, namely P(30), P(28) and P(26). Due to their high intensity, up to 3∙10^−22^ cm/mol, and to an empty-cavity finesse of 100000, low pressures were adopted, from 0.2 to 0.4 Pa depending on the line, to keep the linear absorption below 60%.

On the other hand, the optical power was reduced to 0.5 μW at the cavity output to minimize power broadening of the lines while maximizing the precision of the transition frequency determination. In such conditions the saturation parameter remains around unity (calculation according to ref. 42). Lower power values have not been adopted to prevent the signal-to-noise ratio from being degraded, whereas higher power values would have turned the regime into strong saturation. This regime offers the chance to accurately determine line saturation intensities, yet its exploration remained outside the scope of the paper. The trade-off between SNR degradation and strong saturation effects explains why the investigation has been here limited to the less intense transitions of the 10100-00000 band of acetylene. Actually, the operating conditions used for these lines represent a sort of boundary for the best performance of the spectrometer, which better lends itself to the saturation of weaker transitions, also associated with more symmetric vibration modes and lower electric dipole moments.

**Figure 3:**
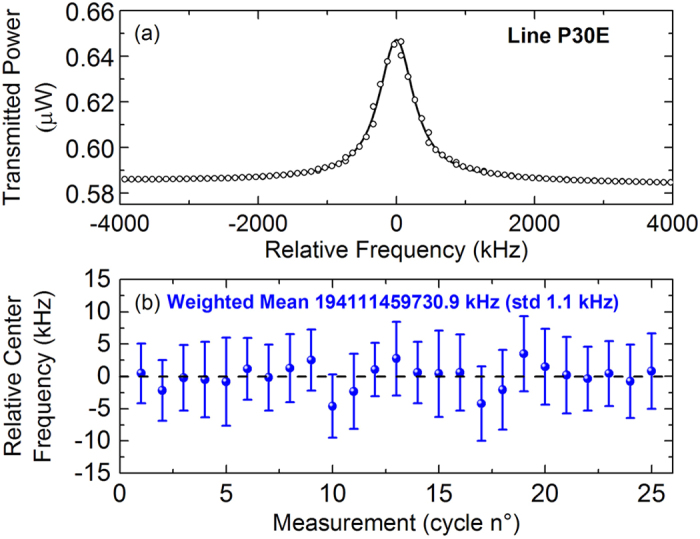
(**a**) 100-fold averaged Lamb dip profile of the P(30) line of the 10100-00000 band of C_2_H_2_ together with a Lorentzian fit with free parameters. (**b**) Scatter of centre frequencies for 25 independent sets of measurements, each one composed of 100 consecutive Lamb-dip spectra. Error-bars report, for each set, the rms deviation of centre-frequencies resulting from the fit of each dip to a Lorentzian profile.

Figure 3(a) reports an averaged Lamb-dip profile for the P(30) line, as obtained at a pressure of 0.4 Pa from a set of 100 consecutive scans over 8 MHz, with 100 spectral points and an overall acquisition time of 200 s (3.3 minutes). The dip closely follows a Lorentzian function and exhibits a contrast of 11% against the saturated absorption measured at the side of the dip. The full-width at half-maximum linewidth retrieved from a Lorentzian fit is 620 kHz: this closely matches the sum of a transit-time contribution^36^ of 230 kHz deriving from an intra-cavity beam-waist radius of 470 μm (50-cm-long cavity with 1-m radius-of-curvature mirrors), a collisional contribution of 220 kHz calculated from a 0.094 cm^−1^/atm broadening coefficient (HITRAN source^4^) and a power-broadening contribution of 180 kHz from a saturation parameter of 0.94. The latter comes as the ratio of an intra-cavity field intensity of 12.7 W/cm^2^, which was calculated dividing the power transmitted from the cavity by the experimental mirror transmission (1.1∙10^−5^), and a saturation intensity of 13.5 W/cm^2^, the latter calculated according to Eq. (22) from ref. 42.

The analysis of the line centre-frequencies retrieved from the fit of the individual dip profiles acquired consecutively within a set of 100 scans, reveals a scattering of about 6–7 kHz rms, depending on the set. This is consistent with a signal-to-noise ratio of nearly 100 that derives from a noise at the 10^−3^ level (see Fig. 2) on a dip with 11% contrast with respect to quite a strong linear absorption greater than 50%. The corresponding precision is 3 parts over 10^11^ over 2 s, which is worse than the 4 parts over 10^12^ that can be attained with an optimized dither-based laser-to-the-dip locking^18^ on the same time window, but more than a decade better than what can be obtained in a comb-assisted Doppler broadening regime^43^,^44^. As compared to dip-locked spectrometers, the main advantage of our approach is the full access to the dip profile, enabling a quantitative study of effects related to collisions, transit-time, saturation, sub-Doppler line-shapes. Figure 3(b) reports the line-centres retrieved from the fit of 25 fully independent sets of measurements, each one encompassing 100 scans, as performed over few days taking care before each set of resetting all locking servos, emptying and refilling the cavity. The rms deviation of data amounts to 1.1 kHz only, which accounts for an impressive Type A uncertainty of 5 parts over 10^12^. The error bars in Fig. 3(b) provide the rms scatter of centre frequencies within a given set, corresponding to the 6–7 kHz value discussed above.

Table 1 summarizes and quantifies the major sources of systematic frequency uncertainties. The stability of the GPS-based frequency standard affects the line position over 200 s by about 0.19 kHz. The 15% accuracy of the absolute pressure gauge used in the experiments translates into a final frequency error of ∼0.18 kHz by considering −3 kHz/Pa as a pressure-shift coefficient: this derives from ref. 45, where the dependence of the pressure shift on the rotational number of the upper quantum state is accounted for. The pressure-induced shift of the line centre is responsible for a further uncertainty contribution of 0.25 kHz over each measurement set due to the relatively high leakage of the cavity, equal to about 1.5 Pa/h. Actually, this relatively high leakage prevented us from prolonging the duration of these sets far above 200 s, thus mitigating the advantage of a highly stable frequency scale and of an inverse-square-root behaviour of the baseline noise upon increasing the number of averaged spectra. The influence of electronic offsets in the PDH lock is negligible since with a PDH discriminator of 310 V/kHz^40^, typical offsets drifts by few mV are not appreciable. A systematic shift was found to originate in our case by a drifting offset between comb and cavity-locked laser during the scan (both having a linewidth of about 10 kHz over 1 ms^40^,^46^). Due to the relatively loose lock between these two signals, a mutual excursion by nearly 200 Hz may happen during the scans, thus adding a further 0.2 kHz term to the uncertainty budget. A 20% uncertainty on the intra-cavity power, as weighted with a power shift coefficient of −11 Hz/mW^47^, gives an almost negligible contribution <0.1 kHz. An uncertainty contribution less than 1 kHz is in the end ascribed to asymmetric residuals in the dip fit, that cannot be accounted for with a purely symmetric Lorentzian function. The quadrature sum of the various systematic shifts leads to a type B standard uncertainty of 1.09 kHz and to an overall uncertainty of ∼1.5 kHz with the quadrature addition of the Type A contribution (1.1 kHz).

Table 2 compares the line-centre frequencies retrieved for the P(30), P(28) and P(26) lines with data determined by Madej *et al*.^18^ at the Canadian metrological institute, which exhibit, amongst the published values, the lowest uncertainties. These have been determined with a cavity of much lower finesse (<400) yet with similar intra-cavity field intensities at a pressure of 2.7 Pa through the typical top-of-the-dip locking approach adopted for frequency standards. The comparison requires quite a severe correction by more than 6 kHz for the pressure shift, due to the use in our case of nearly 10-times lower pressure values (0.4 Pa for the P(30), 0.2 Pa for the P(28) and P(26) lines): this correction has been reported with a + sign in Table 2 to make frequencies from Madej *et al*. (higher pressure) comparable with ours. A second correction term with opposite sign has been introduced to account for the effect of a rather large 1.8-MHz peak-to-peak modulation depth adopted in ref. 18 for dip locking: the same authors report for this effect a shift coefficient of −4.7 kHz/MHz_pp_^47^, which translates into a final correction by −8.5 kHz due to the absence, in our case, of any modulation. Interestingly, despite the very different working conditions and the strong impact of both pressure and amplitude modulation effects, the offset between the two frequency sets stays within the respective uncertainties, in both cases reported at the 2-kHz level only. The offset amounts to roughly 2.6 kHz with a sub-kHz dispersion, thus attesting a very high precision for both approaches. The comparison is unfortunately limited to three lines due to the difficulty, in our setup, to preserve the same level of accuracy for the much more intense lines that occur with lower rotational quantum numbers of the upper state. On the other hand, it emerges from Table 2 that one of the main hurdles in the traceability and eventually in the accuracy of spectroscopic determinations derives from the impact that different working conditions may have on the systematic errors: with synchronous detection schemes the impact given by the modulation depth, if not properly accounted for, may significantly impair the comparison between different determinations. Moreover, if the dip-profile is not measured but simply used as a marker for a top-of-fringe locking, one may miss the impact of profile asymmetries on the final line center value^48^. With our approach, instead, the cavity is not modulated and the frequency axis is fully defined by the comb at every point of the dip profile, thus making possible to catch rather subtle effects that do not emerge at the output of a lock-in amplifier. A further strength of the approach is given by the possibility to work at very low pressures, thus with smaller linewidths and lesser impact from pressure-induced shifts that cannot be easily accounted for in the absence of high-quality spectroscopic data. This is for example the case of HITRAN, which reports for the whole band a single pressure shift value of −1 kHz/Pa.

**Figure 4:**
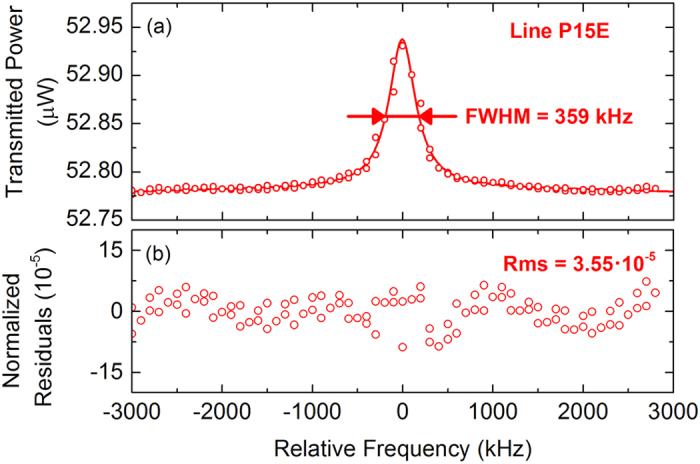
(**a**) 100-fold averaged Lamb-dip profile of the P(15) line of the 01120-00000 band at a pressure of 0.2 Pa. (**b**) Lorentzian fit residuals, as normalized to the empty-cavity transmission (56 μW).

To test the ability of the spectrometer to saturate much weaker transitions we moved to the P(15) line of the 01120-00000 band. The line strength is 9.468∙10^−24^ cm/mol and gives rise to a linear absorption of about 10% at the adopted pressure of 0.18 Pa. The Einstein coefficient of the transition is 5.632 mHz, i.e. three orders of magnitude lower than those of the 10100-00000 band and close to the smallest ever explored in a saturated regime^36^. To saturate the transition the power level at the cavity output has been raised to a level of ∼50 μW, corresponding to an intra-cavity field-intensity of ∼1.5 kW/cm^2^. In such conditions the dip was clearly visible, as shown by the 100-fold-averaged Lamb-dip spectrum reported in Fig. 4. The dip contrast is 0.3%, thus in rather good agreement with an expected saturation parameter of 0.08. In such conditions the linewidth is negligibly affected by power broadening and amounts to 359 kHz only. The residuals of a Lorentzian fit were found to be limited by a non perfect line shape model and by a non perfect baseline to a rms normalized value of 3.55∙10^−5^. Remarkably, this is nearly an order of magnitude below that given by the Allan variance plot of Fig. 2 and resulted from an extremely careful optimization of the setup and suppression of ground loops that was found hardly reproducible, which is the reason why the discussion above has been set on the normal performance regime of the spectrometer. However, those residuals are a demonstration that a sensitivity limit of 3.1∙10^−12^ cm^−1^ over 200 s can be achieved with a direct detection approach. In such conditions the statistics on the line centre frequencies retrieved from the fit of 100 consecutive scans is characterized by a scattering of 18–24 kHz, thus only a factor of 3–4 worse than for the 10100-00000 band notwithstanding a 1000-times lower Einstein coefficient. The systematic errors are negligibly affected by the different band and line, whereas statistical errors are averaged down to 5 kHz only, which results in a centre-frequency of 192248852.795(5) MHz at a pressure of 0.2 Pa.

## Conclusions

This paper describes a versatile comb-assisted cavity-enhanced system for Lamb-dip spectroscopy at a variety of wavelengths in a rather large spectral range from 1.5 to 1.63 μm. It relies on two servo loops only, one for the laser-to-the-cavity locking, the other for the cavity-to-the-comb locking. Detection is performed directly at the cavity output with high signal-to-noise ratio thanks to an extremely tight Pound-Drever-Hall (PDH) locking based on an integrated single-sideband modulator. Thanks to the high finesse of the cavity and to an intra-cavity field intensity that can be varied over two decades overcoming the kW/cm^2^ threshold, the system lends itself to the saturation of a variety of lines never saturated so far, also including weak ones. From a spectroscopic point of view it may become a workhorse for highly accurate surveys of line centre-frequencies of thousands of transitions, over several absorption bands of a variety of molecules, as well as for the investigation of line-shape models and saturation effects in a Doppler-free regime. From a technological point of view, the current sensitivity at the 10^−11^ cm^−1^ level may be further improved by minimizing the cavity leakage, which currently bounds the averaging time to few hundreds of seconds. A leap forward would be possible by arranging the system in a NICE-OHMS configuration. A vast deployment of NICE-OHMS has been prevented so far by the difficulty in achieving tight PDH locking with conventional broadly tunable cw lasers. The single-sideband modulation scheme demonstrated here removes this stumbling block and paves the way to shot-noise limited spectroscopy at the output of high-finesse cavities.

## Additional Information

**How to cite this article**: Gatti, D. *et al*. Comb-locked Lamb-dip spectrometer. *Sci. Rep*. **6**, 27183; doi: 10.1038/srep27183 (2016).

## Figures and Tables

**Table 1:** Summary of estimated systematic uncertainties associated with the absolute measurement of line centre frequencies.

Sources of systematic errors	Estimated shift [kHz]
Frequency reference	0.19
Pressure measurement	0.18
Pressure leakage	0.25
Comb-cavity lock point	0.2
Power shift	0.1
Spectral profile asymmetry	1
**Total shift**	**1.09**

**Table 2:** Comparative analysis between this work and Madej *et al*. 18.

Line	Freq. [kHz]	Uncert. [kHz]	Press. correct. [kHz]	Modul. correct. [kHz]	Corrected Freq. of ref. 18 [kHz]	Uncert. of ref. 18 [kHz]	Deviation [kHz]
P(30)	194111459730.9	1.5	6.9	−8.5	194111459733.4	1.9	−2.5
P(28)	194295440625.8	1.8	8.1	−8.5	194295440629.1	2	−3.4
P(26)	194476488862.0	1.9	8.1	−8.5	194476488864.0	2.4	−2.1

Measured absolute frequencies for the P(30), P(28), P(26) lines of the (10100) ← (00000) band of C_2_H_2_ (at 0.4, 0.2 and 0.2 Pa respectively) as compared to those from ref. 18 (2.^7^ Pa): these have been corrected by the pressure and amplitude-modulation shifts reported in the Table through the coefficients measured in refs 45 and 47, respectively. The uncertainty refers for both cases to the quadrature addition to Type A and Type B errors. The deviation between the two frequency sets is calculated by subtracting data from Madej *et al*. to our values.
